# A rare case of prostate cancer initially presented by disseminated intravascular coagulation‐related subdural hemorrhage

**DOI:** 10.1002/cnr2.1868

**Published:** 2023-07-12

**Authors:** Mahan Shafie, Elnaz Shahmohamadi, Alireza Hadizadeh, Alireza Hasanzadeh, Golsa Gholampour, Samaneh Parsa

**Affiliations:** ^1^ School of Medicine Tehran University of Medical Sciences Tehran Iran; ^2^ Department of Internal Medicine Imam Khomeini Hospital Complex, Tehran University of Medical Sciences Tehran Iran

**Keywords:** disseminated intravascular coagulation (DIC), prostate cancer, subdural hemorrhage (SDH)

## Abstract

**Background:**

Disseminated intravascular coagulation (DIC) has been reported in various solid malignancies and is a common coagulation‐related complication in prostate cancer. However, DIC has been rarely reported as the initial presentation of prostate cancer. Herein, we reported a patient referring with subdural hemorrhage (SDH) and DIC with an unexplained cause who was later diagnosed with prostate cancer.

**Case Presentation:**

We presented a 68‐year‐old man who was referred to the hospital with a gradual deterioration of consciousness, dyspnea, and edema in the genitalia and lower limbs. His primary laboratory tests showed elevated prothrombin time (PT) and partial thromboplastin time (PTT) and a decreased fibrinogen level of 47 mg/dL [200–400 mg/dL]. The DIC score was 7, which was suggestive of DIC. Moreover, cranial imaging showed SDH. Further work‐up revealed elevated prostate‐specific antigen and prostate enlargement with a mass effect on the bladder with a bone lesion, which was suggestive of metastatic prostate cancer.

**Conclusion:**

This report highlights DIC as a possible initial presentation of an underlying malignancy, as well as the importance of treatment of underlying disease in the management of DIC. A comprehensive and systematic work‐up is essential for early diagnosis in patients with DIC to avoid further complications and mortality.

## 1 INTRODUCTION

Disseminated intravascular coagulation (DIC) is a life‐threatening coagulation disorder that may occur in a wide range of clinical conditions. A comprehensive exploration should be performed for any underlying medical disorder, including sepsis, trauma, solid and hematological malignancies, obstetrical complications, and vascular disorders after confirmation of the DIC diagnosis.^1^ DIC has been reported in different solid malignancies and is a coagulation‐related complication of prostate cancer.^2^ The incidence of DIC in patients with prostate cancer is estimated between 13% and 30%.^2^ Although DIC is a coagulopathy that often complicates prostate cancer, it has been rarely reported as the initial presentation of prostate cancer.^3^, ^4^, ^5^

Herein, we reported a 68‐year‐old man who presented with a gradual deterioration of consciousness, dyspnea, and lower limb edema who later was discovered to have DIC and subdural hemorrhage (SDH) as an uncommon initial presentation of prostate cancer. This report highlights DIC as it could be a primary manifestation of an underlying malignancy, as well as the importance of early diagnosis and treatment of underlying disease in the management of DIC. Due to the high mortality of DIC in a patient with an underlying malignancy, a comprehensive and systematic work‐up, and management is critical to avoid further complication and mortality.

## 2 CASE PRESENTATION

In November 2021, a 68‐year‐old man with a past history of hypertension presented to the emergency department of our center affiliated with Tehran University of Medical Sciences with a gradual deterioration of consciousness, shortness of breath, and also symptoms of volume overload, which included edema in lower limbs, especially in the right lower limb, as size difference was clearly observed. He had also experienced dizziness and fall incidents during the last few months, but reported no head injury. He denied having a history of abdominal pain, fever, nausea, vomiting, urinary symptoms, or significant weight loss. He denied the use of any medications except for hypertension. His habitual history was negative, and he did not report a family history of cancer or hematologic disorders. The vital signs showed tachycardia (105 bpm), tachypnea (23 rpm), and fever (38.1°C); his blood oxygen saturation was also low (77%). His physical examination showed severe edema (level 3) on lower limbs, genitalia, and also upper limbs. His abdomen was soft, without tenderness or hepatosplenomegaly; however, moderate ascites were notable. Several bruising and ecchymosis were noted on the back and lower limbs. Upon auscultation, no abnormal cardiac sounds were heard, but pulmonary sounds, particularly on the right side, were diminished. The cranial nerve examination and cerebellar tests, including Romberg's test, were normal. No lymphadenopathy was discovered in physical examination.

**FIGURE 1:**
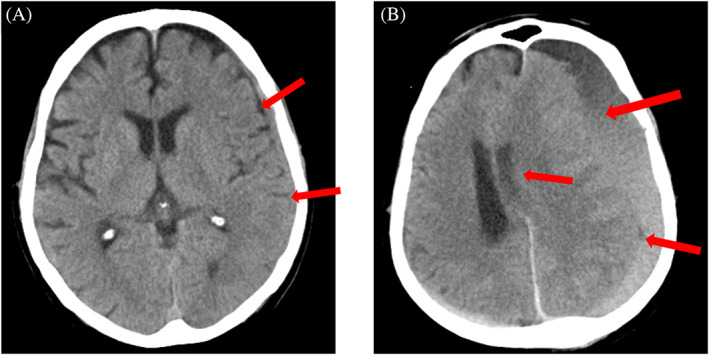
(A) Axial cranial computed tomography (CT) scan obtained upon admission showing subdural hemorrhage (SDH) in the left hemisphere (arrows). (B) Axial cranial CT scan obtained during hospitalization showing massive SDH and midline shift (arrows).

**FIGURE 2:**
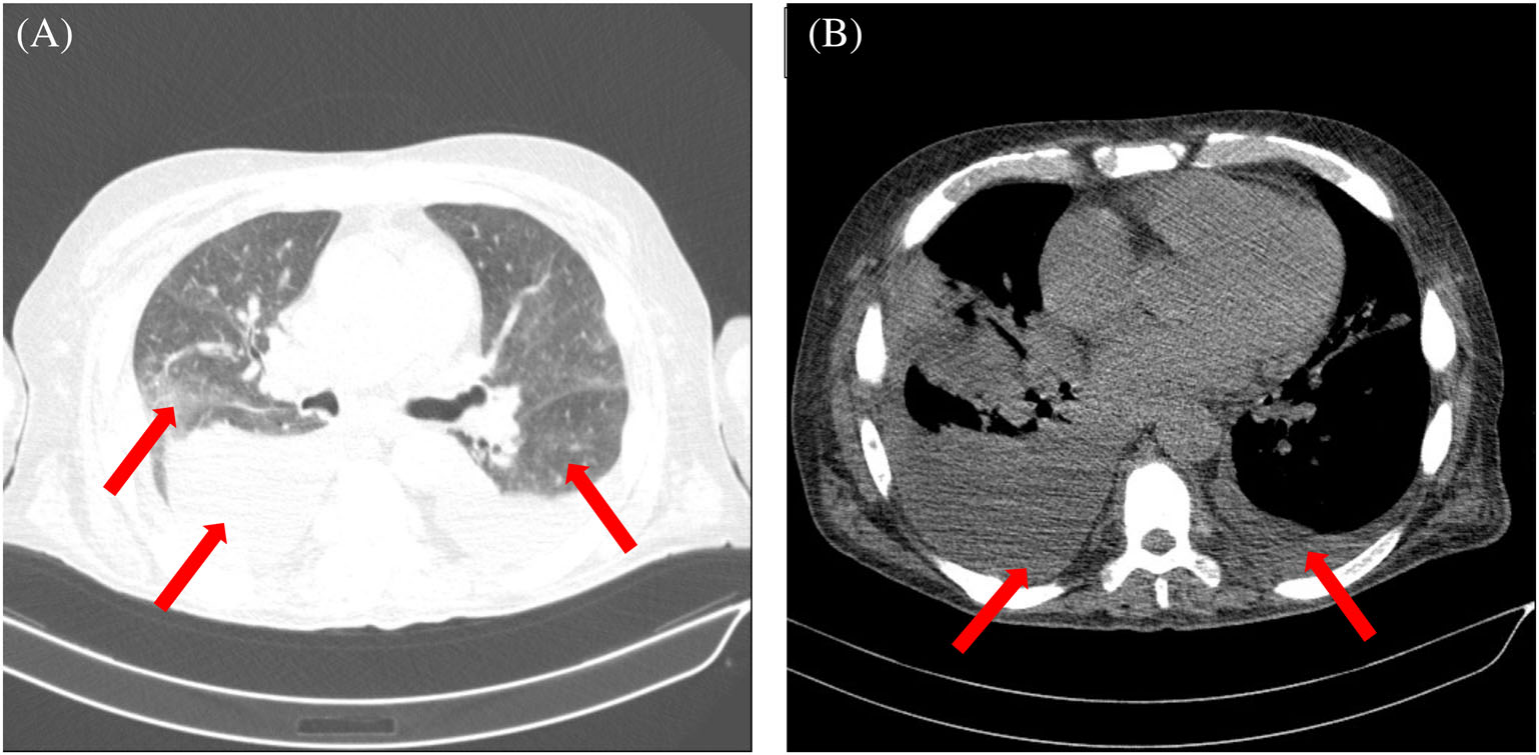
(A) Axial chest computed tomography (CT) scan obtained at pulmonary window of lower region of the thorax showing ground glass opacity and collapse consolidation (arrows). (B) Axial chest CT scan obtained at thoracic window of lower region of thorax showing pleural effusion (arrows).

The laboratory data of the patient showed hemoglobin of 8.6 g/dL [normal range: 14–17 g/dL], white blood cell count of 7.7 × 10^3^/mm^3^ [4.5–11.0 × 10^3^/mm^3^], and platelet count of 50 × 10^3^/mm^3^ [150–400 × 10^3^/mm^3^]. Liver function tests revealed elevated alkaline phosphatase along with slightly increased levels of aspartate aminotransferase and bilirubin. A peripheral blood sample revealed schistocytes and hyper‐segmented neutrophils. The coagulation test revealed the following results: A prothrombin time (PT) of 26 s [11–13.5 s], a partial thromboplastin time (PTT) of 60 s [30–40 s], a thrombin time of 29.6 s [14–21 s], an international normalized ratio (INR) of 2.2 [0.0–1.1], a fibrinogen level of 47 mg/dL [200–400 mg/dL], and fibrinogen degradation product (FDP) of 160 μg/mL [0–5 μg/mL] (Table 1). The DIC score was 7 based on the International Society of Thrombosis and Hemostasis diagnosis criteria.^6^ The patient was diagnosed as having overt DIC and treatment with cryoprecipitate and fresh frozen plasma was initiated immediately. Cranial and thoracic computed tomography (CT) scans, and also abdominal sonography were requested. The cranial CT scan revealed mild SDH (<1 mm) in the left cerebral hemisphere, while the consultation with the neurosurgical department indicated the patient did not require surgical intervention (Figure 1A). Meanwhile, a chest CT scan revealed bilateral severe pleural effusion (more severe on the right) and collapsed consolidation on both sides, which was suggestive of bacterial pneumonia (Figure 2). The sonographic evaluation showed free fluid in the abdomen, and although no pathology within the parenchyma of the liver or spleen was recorded, the inferior vena cava and hepatic veins were markedly dilated. A color doppler sonography of the lower limb vessel was also performed, which was negative for deep vein thrombosis. According to the findings, along with an echocardiographic assessment, we ruled out pulmonary thromboembolism with a ventilation‐perfusion scan. The evaluation was furthered after the correction of coagulopathy, using pleural fluid tapping, which showed that the pleural effusion was exudative, and subsequently, antibiotic therapy with levofloxacin was initiated (Table 2). Echocardiography revealed increased pulmonary artery pressure (LVEF: 55%–60%, PAP: 44 mm Hg), which confirmed the suspected preserved ejection fraction heart failure.

**TABLE 1:** Laboratory data.

Laboratory test	Findings	Normal range	Laboratory test	Findings	Normal range
WBC	7.7	4.5–11.0 × 10^3^/mm^3^	ALT	35	4–36 unit/L
Hb	8.6	14–17 g/dL	ALP	1768	44–147 unit/L
Platelet	50	150–400 × 10^3^/mm^3^	Bilirubin total	1.3	<1.2 unit/L
Na	139	135–145 meq/L	Bilirubin direct	0.6	<0.3 unit/L
K	4.6	3.6–5.2 meq/L	Cr	1.7	0.7–1.3 mg/dL
Ca	7.9	8.5–10.2 meq/L	Urea	72	6–24 mg/dL
P	3.2	2.8–4.5 meq/L	Uric acid	9.4	3.5–7.2 mg/dL
Mg	2	1.7–2.2 meq/L	LDH	748	105–333 unit/L
pH	7.48	7.35–7.45	Albumin	3.5	3.4–5.4 g/dL
pCO_2_	32	35–45 mmHg	Total protein	5.8	6–8.3 g/dL
HCO_3_	24	22–28 meq/L	Retic count	7.9	0.5–2.5%
PT	26	11–13.5 s	Corrected retic count	5.4	0.5–2%
PTT	60	30–40 s	CRP	40	<0.9 mg/L
INR	2.2	<1.1	ESR	5	0–22 mm/hour
Fibrinogen	47	200–400 mg/dL	Ferritin	311	24–336 ng/mL
FDP	160	0–5 μg/mL	HBS Ag	Non‐reactive	
D dimer	7333	<500 ng/mL	Anti‐HCV	Non‐reactive	
PSA	12 (ng/mL)	<4 ng/mL	HIV Ag/Ab	Non‐reactive	
AST	42	8–33 unit/L			

Abbreviations: ALP, alkaline phosphatase; ALT, alanine aminotransferase; AST, aspartate aminotransferase; Cr, creatinine; CRP, c‐reactive protein; ESR, erythrocyte sedimentation rate; FDP, Fibrin degradation products, PSA, prostate‐specific antigen; Hb, hemoglobin; INR, international normalized ratio; LDH, lactate dehydrogenase; PT, prothrombin time; PTT, partial thromboplastin time; WBC, white blood cell.

**TABLE 2:** Pleural fluid analysis.

Laboratory test	Findings (unit)	Laboratory test	Findings (unit)
WBC	44 (mm^3^)	Glucose	91 (mg/dL)
Poly	36 (mm^3^)	LDH	223 (unit/L)
Lymph	64 (%)	WBC	0–1 (HPF)
RBC	500 (%)	RBC	1–2 (HPF)
Albumin	1800 (mg/dL)	Bacteria (Gram Stain)	Negative
Protein	2700 (mg/dL)	Culture	Negative

Abbreviations: LDH, lactate dehydrogenase; RBC, red blood cell; WBC, white blood cell.

**FIGURE 3:**
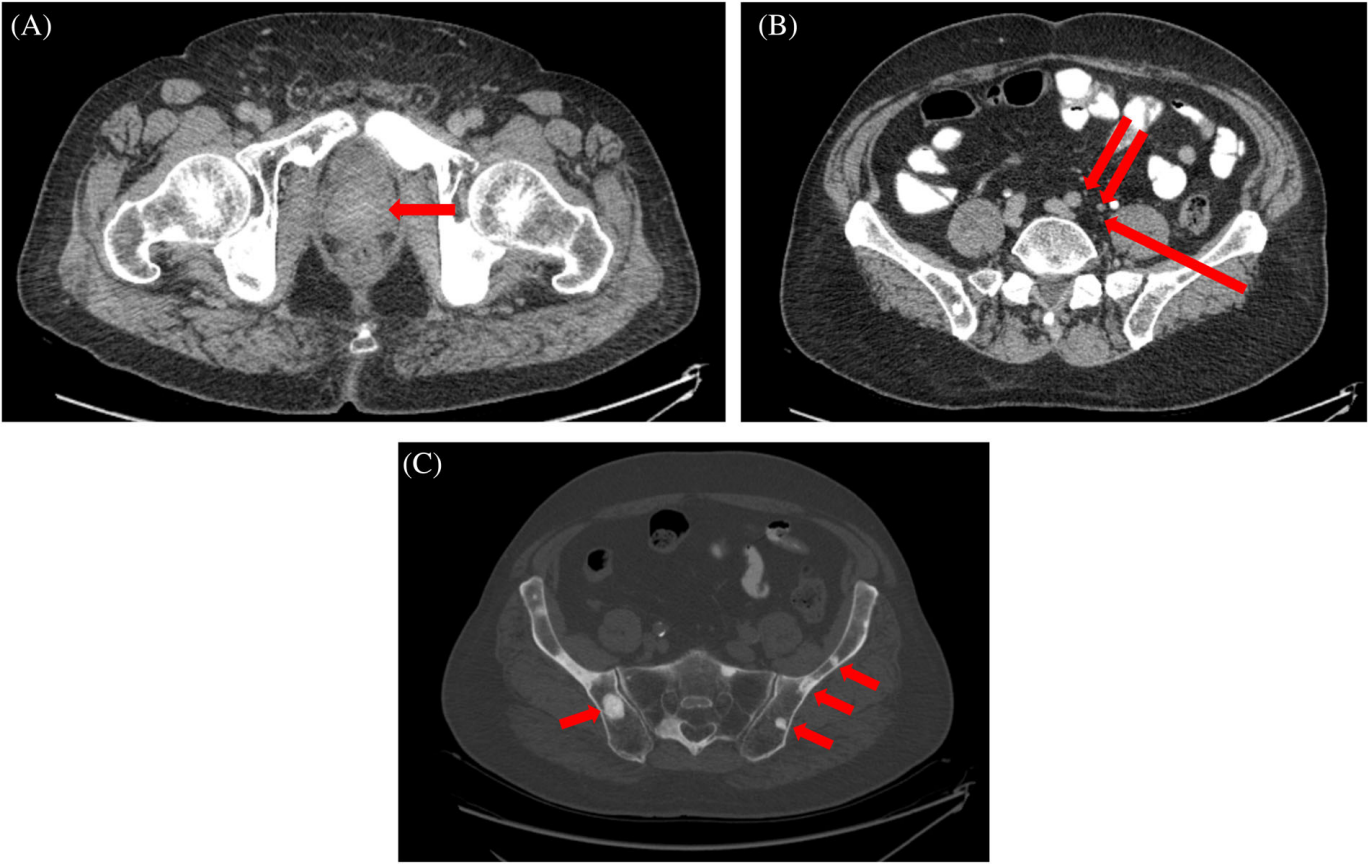
(A) Pelvic computed tomography scan showing prostate (arrow), (B) para‐aortic lymphadenopathies (arrows), and (C) metastatic pelvic bone lesions (arrows).

A search to determine the underlying etiology was continued and a comprehensive malignancy work‐up was performed. Serum prostate‐specific antigen (PSA) was 12 ng/mL (normal: <4 ng/mL). Prostatic induration was identified through the digital rectal exam. Abdominal ultrasound investigation reported enlarged prostate with a volume of about 70 cc with a mass effect on the bladder. Increased distended bladder wall thickness was also noted. The patients underwent an abdominopelvic CT scan without contrast, which revealed a prostate enlargement (57 × 57 × 44 mm) with two hypo‐signal nodules in the right middle and left inferior portions of the inner gland, along with multiple left para‐aortic, left external and internal iliac, and left peri‐rectal lymphadenopathies (Figure 3). Further investigations also revealed bone metastases, specifically in the pelvic bone, however, no liver metastasis was observed. As the patient's condition became stable, a prostate biopsy was also performed, which reported prostate adenocarcinoma with a Gleason score of 8 and grade group of 4 with perineural invasion. Based on the above findings, a diagnosis of metastatic prostate cancer was confirmed in the patient. Urology and oncology consultation was requested accordingly, and medical androgen deprivation therapy (ADT) including triptorelin and bicalutamide was recommended for the patient. Four days after the diagnosis confirmation, the patient lost consciousness, and another brain CT scan showed that SDH had exacerbated (Figure 1B). As there was a clear midline shift, immediate acute care with surgical intervention and craniotomy along with mannitol fluid therapy was performed, and the patient was transferred to the intensive care unit (ICU). Unfortunately, the symptoms exacerbated, resuscitation and cardiopulmonary resuscitation (CPR) were unsuccessful, and the patient expired.

## 3 DISCUSSION

This study reports a case presented to the emergency department with dyspnea and signs of volume overload. His evaluations revealed mild SDH and pleural effusion, which turned out to be exudative. During the course of hospitalization, the patient's DIC was exacerbated, and subsequently developed massive SDH.

DIC is conventionally characterized by prolonged PT and PTT, low fibrinogen, increased FDPs and D‐dimer, and low platelet count. Lower fibrinogen levels usually indicate a more severe DIC, but, as fibrinogen is an acute‐phase reactant, serial measurements can be a more reliable indicator.^7^ International Society of Thrombosis and Hemostasis has set the criteria by which DIC could be identified.^6^ According to this criteria, a score greater than or equal to five is indicative of DIC (Table 3).

**TABLE 3:** International society of thrombosis and hemostasis scoring system for disseminated intravascular coagulation.

Laboratory test	Result	Score
Platelet count	>100 × 109/L	0
	<100 × 109/L	1
	<50 × 109/L	2
Elevated fibrin‐related marker (FDP/D‐dimer)	No increase	0
	Moderate increase	2
	Strong increase	3
Prothrombin time prolongation	<3 s	0
	>3 s but <6 s	1
	>6 s	2
Fibrinogen level	>100 mg/dL	0
	<100 mg/dL	1

DIC occurs due to widespread activation of coagulation pathways, characterized by thrombotic occlusion of blood vessels, which can compromise organ blood supply, and at the same time, increased the probability of bleeding secondary to consumption of coagulation factors and platelets. DIC usually occurs secondary to an underlying cause, including severe trauma, severe infectious disease, obstetric disorders, immunological disorders, reactions to toxins, and hematological and solid malignancies.^8^ Therefore, it is important to search for underlying conditions in cases with no apparent cause.^7^ In the current report, there were a number of differential diagnoses including thrombotic microangiopathy (TMA) such as hemolytic uremic syndrome (HUS) and thrombotic thrombocytopenic purpura (TTP), sepsis especially due to gram‐negative bacteria, congenital coagulopathies, liver disease, vascular malformations such as aneurysms and hemangiomas, and trauma. TMA was ruled out due to the presence of coagulopathies. Sepsis was ruled out due to negative cultures and negative systemic inflammatory response syndrome (SIRS) and sequential organ failure assessment (SOFA). There was no history of congenital coagulopathies and no evidence of liver disease and vascular malformation was obtained during the laboratory and radiologic workup. Despite the history of dizziness and fall incidents, no sign of head trauma was reported.

The association between DIC and malignancies has been well documented by potential mechanisms such as procoagulants secreted by malignant cells.^9^, ^10^ Acute or chronic DIC are coagulation‐related complications of prostate cancer, and the incidence of subclinical DIC might range between 24% and 40% in metastatic prostate cancer patients.^11^ Nevertheless, the pathophysiology linking prostate cancer and DIC is not fully understood.^12^ According to previous studies, the expression of procoagulant molecules from tumor cells, notably tissue factor, activates the host's hemostatic system, resulting in thrombosis and the consumption of coagulation factors, which ultimately leads to DIC.^13^ Besides, previous studies showed that patients with prostate cancer complicated by DIC had a very poor prognosis, and therefore, intensive management and active cancer therapy might improve overall survival in this population.^14^ However, developing DIC as a first presentation of prostate cancer has been rarely reported.^3^, ^15^, ^16^

DIC in prostate cancer can become complicated with either spontaneous hemorrhage in various sites or thrombotic events. The presentations of bleeding vary from mild petechia and ecchymosis to severe gastrointestinal or intracranial bleeding, while thrombotic events could cause venous or arterial thromboembolism in multiple organs and result in ischemia. Even though complications of DIC could be very serious, DIC is rarely symptomatic in prostate cancer and is present in only 0.4%–1.65% of patients, and most of the patients remain clinically asymptomatic.^17^ Chronic DIC is a common presentation in patients with malignancies. It results from a persistent weak or intermittent coagulation‐activating stimulus. We assume that our patient suffered from this phenomenon as his impaired coagulation tests and mild SDH suggests.^18^ Spontaneous intracranial hemorrhage can occur in patients with malignancies due to various etiologies, while the most common cause is DIC.^19^ A study conducted by Navi et al assessed 208 known cancer subjects who also developed intracranial hemorrhage. This study concluded that coagulopathy and intra‐tumoral hemorrhage constitutes a majority of the hemorrhages, while hypertension plays a minor role.^20^

The main etiology for SDH is a head injury, however, non‐traumatic pathophysiologies like vascular malformations, neoplasms, aneurysms, and coagulopathy can be the cause. In this case, a brain CT scan upon admission revealed SDH, suspected to be a consequence of chronic DIC considering the laboratory tests, but other etiologies could not be completely ruled out. The patient reported experiencing fall incidents due to dizziness but none of them was associated with trauma to the head. Moreover, no sign of head trauma and hematoma of the skull was reported in the physical evaluation. Other bleeding conditions that can lead to intracerebral hemorrhage in cancer patients include liver metastases, which decrease coagulation factors, and thrombocytopenia caused by tumor invasion of the bone marrow or radiation and chemotherapy's effects on the bone marrow.^19^ Yet, no liver metastasis was detected in the presented patient.

The association between DIC and intracranial hemorrhage and various cancers has been well documented.^19^ Claes et al. reported a case that was previously diagnosed with prostate cancer 2 years prior to presentation to the emergency department with left facial palsy and confusion. He was also diagnosed with intracerebral hematoma due to DIC.^21^ However, there are only a few studies reported intracranial hemorrhage secondary to DIC as an initial presentation of prostate malignancy. In a case reported by Kojima et al,^22^ a patient was referred with a sudden onset of consciousness disturbance and right hemiplegia. This patient was diagnosed with intracranial hemorrhage secondary to DIC, and the following workup revealed occult prostate cancer. Johno et al also described a case of intracerebral hemorrhage as the initial presentation of DIC associated with underlying prostate cancer.^23^

The primary approach to the management of DIC is to approach the underlying cause. The management also involves appropriate supportive care for the impaired coagulative state. Life‐threatening conditions such as excessive hemorrhage could necessitate the transfusion of fresh frozen plasma, cryoprecipitate, and platelets along with the administration of antifibrinolytic agents such as tranexamic acid or epsilon aminocaproic acid. In cases with thrombosis‐predominant DIC, heparin in therapeutic doses should be considered.^24^ Moreover, hormonal therapy, chemotherapy, and radiopharmaceutical therapy are reported to be effective treatment choices for patients with DIC related to prostate cancer.^17^ In our patient, treatment of DIC was initiated immediately after the diagnosis confirmation. Right after the correction of the patient's coagulopathy, further work‐up for the underlying condition was performed, and the diagnosis of prostate cancer was confirmed within a few days. Since studies have suggested that resolving the underlying disorder could prevent further exacerbation of DIC, ADT was initiated for the patient right after confirmation of cancer diagnosis. While our patient was under supportive care with cryoprecipitate and fresh frozen plasma, his SDH expanded, and the care could not prevent the exacerbation of hemorrhage. Regarding therapeutic agents for DIC, it should be noted that a large amount of FFP takes longer to infuse, while prothrombin concentrate complex (PCC) can be administered over a few minutes and provides immediate reversal in life‐threatening bleeding, which could be one of our limitations in the management of the disease. The International Prognostic Index (IPI) evaluates multiple factors such as age and stage of a disease and hence provides a score by which the survival rate is assessed. Using this method, we calculated a score of 5, which shows a low survival rate even if the patient survived DIC and SDH.^25^, ^26^, ^27^

## 4 CONCLUSION

This case highlights the fact that patients with prostate cancer could initially present with intracranial hemorrhage secondary to DIC. A comprehensive and systematic work‐up is essential for early diagnosis in patients with DIC to avoid further complications and mortality. Due to the high mortality rate of DIC in an underlying malignancy, early diagnosis of the underlying cause and intensive management could be life‐saving for patients.

## AUTHOR CONTRIBUTIONS

**Mahan Shafie:** Conceptualization (equal); writing – original draft (equal); writing – review and editing (equal). **Elnaz Shahmohammadi:** Writing – original draft (equal); writing – review and editing (equal). **Alireza Hadizadeh:** Conceptualization (equal); writing – original draft (equal); writing – review and editing (equal). **Alireza Hasanzadeh:** Writing – original draft (equal); writing – review and editing (equal). **Golsa Gholampour:** Writing – review and editing (equal). **Samaneh Parsa:** Conceptualization (equal); supervision (equal); writing – review and editing (equal).

## CONFLICT OF INTEREST STATEMENT

The authors have stated explicitly that there are no conflicts of interest in connection with this article.

## ETHICS STATEMENT

This study was approved by the research and ethics committee of Tehran University of Medical Sciences.

## INFORMED CONSENT

Written informed consent was obtained from the patient's next of kin for publication of the case report. A copy of the written consent is available for review by the Editor‐in‐Chief of this journal.

## Data Availability

The data used to support the findings of this study are available from the corresponding author upon request.
